# Inflammation and Cognitive Functioning in Older Adults: The Role of Gender and Obesity

**DOI:** 10.1093/geroni/igab046.2606

**Published:** 2021-12-17

**Authors:** Melody Moloci Noss, Summer Millwood, Kate Kuhlman

**Affiliations:** 1 University of California, Irvine, Irvine, California, United States; 2 University of Denver, Denver, Colorado, United States

## Abstract

Systemic inflammation is associated with steeper cognitive decline over time. Identifying potential moderators of inflammation is crucial for understanding inflammation’s contribution to abnormal cognitive decline. This study examined whether inflammation predicted changes in cognitive functioning over time and explored the moderating effects of sex and BMI on this association. Data was collected from a longitudinal nationally representative data set. (Health & Retirement Study). C-reactive protein (CRP) and global cognitive functioning assessments were collected from the 2006/2008 and 2010/2012 waves. Participants, n= 7,483, Age =71.39 years (SD = 9.24) , 60.2% female, were categorized into groups based on BMI (i.e. normal, overweight, and obese). Sex and BMI significantly moderated the association between increased hs-CRP and lower cognitive functioning, b = -.22 (SE = .09), p = .017. Women with high BMI exhibit twice the risk of low cognitive functioning, b = -.49 (SE = .07), p < .0001, compared to men with high BMI, b = -.21 (SE = .08), p = .01. Men with normal BMI exhibited twice the risk of low cognitive functioning, b = -.49 (SE = .08), p < .0001, compared to women with normal BMI, b = -.24 (SE = .06), p = .0001. Inflammation and BMI are modifiable factors that may prevent or slow -down abnormal cognitive decline. Understanding the potentially sex-dependent role of adipose tissue in the impact of inflammation on cognitive function may be critical to understanding the pathogenesis of cognitive impairment late in life as well as identifying efficacious intervention targets.

